# Extended middle meatal antrostomy via antidromic extended medial wall for the treatment of fungal maxillary sinusitis

**DOI:** 10.1186/s12893-022-01739-0

**Published:** 2022-07-25

**Authors:** Lijun Ding, Na-guo, Zhengcai Lou

**Affiliations:** 1 Department of Operating Theater, Yiwu Central Hospital, 699 Jiangdong Road, Yiwu City, 322000 Zhejiang Province China; 2 Department of Science and Education, Qingdao Chengyang People’s Hospital, No. 600 Changcheng Road, Chengyang District, Qingdao, Shandong Province China; 3 Department of Otorhinolaryngology, Yiwu Central Hospital, 699 Jiangdong Road, Yiwu City, 322000 Zhejiang Province China

**Keywords:** Middle meatal antrostomy, Inferior meatal antrostomy, Fungal maxillary sinusitis, Recurrence rate

## Abstract

**Objective:**

The objective of this study was to compare the long-term results of extended middle meatal antrostomy (MMA) and MMA combined with inferior meatal antrostomy (IMA, combined approach) for the treatment of fungal maxillary sinusitis (FMS).

**Methods and materials:**

A retrospective analysis including 90 patients with non-invasive FMS was treated with endoscopic extended MMA via antidromic extended medial wall (extended MMA group), or with both MMA and IMA (combined approach group). The recurrence rate, operation time, and complications were evaluated at postoperative 12 and 36 months.

**Results:**

Of the 90 patients, 52 patients were in the extended MMA group and 38 patients in the combined approach group. CT revealed the thin medial wall or bone defect in 63.33% (57/90) patients. The mean operation time in the extended MMA group was significantly shorter than that of combined approach group (42.5 ± 6.5 vs 57.4 ± 4.9, P < 0.01). At postoperative 12 months postoperatively, the recurrence rate was 3.85% (2/52) in the extended MMA group and 0.0% (0/38) in the combined approach group, the difference wasn’t significant (*X*^*2*^ = 0.618, P > 0.05). The recurrence rate wasn’t increased during the follow-up period over time in both groups.13.5% (7/52) patients complained of cheek numbness in the extended MMA group, 60.5% (23/38) patients complained of cheek numbness and epiphora in 5.3% (2/38) patients in the combined approach group, the difference was significant (*X*^*2*^* test*, P < 0.01). However, no major complications were observed in both groups. In addition, IMA closure was observed in 4 (10.5%) in the combined approach group at 12 months postoperatively and in 9 (23.6%) at 36 months postoperatively.

**Conclusions:**

Extended MMA via antidromic extended medial wall may effectively prevent the recurrence and reduce the complications of FMS, IMA wasn’t necessary for the treatment of FMS in most cases.

## Introduction

Fungal sinusitis is broadly categorised as either invasive or non-invasive, non-invasive fungal sinusitis is subdivided into allergic fungal sinusitis and fungus Ball (FB) [1, 2]. FB is the most frequent cause of non-invasive fungal sinusitis, and the maxillary sinus (MS) is the most common location in China [2]. Fungal maxillary sinusitis (FMS) was once considered a relatively uncommon disease but its incidence has increased dramatically over the last 2 decades [2–4]. The Caldwell-Luc operation was widely used to treat FMS before endoscopic sinus surgery (ESS). At present, ESS has become the accepted treatment [4–6]. However, the standard ESS approach with middle meatal antrostomy (MMA) had high recurrence[4–6], some previous reports have recommended a combined approach of MMA and inferior meatal antrostomies (IMA, combined approach), for severe cases of MS [7–9]. Nevertheless, very few reports have evaluated the results of a combination of MMA with an IMA for FMS, and it has not been established whether IMA is necessary for successful ESS. Furthermore, IMA might harm long-term MS mucociliary clearance [10, 11].

In recent years, we performed the extended MMA via antidromic extended medial wall to treat FMS. The objective of this study was to compare the long-term results between extended MMA and MMA combined with IMA for the treatment of FMS.

## Materials and methods

We retrospectively studied the clinical data and operative records of 90 patients with FMs who underwent surgery from May 2013 to January 2018 in the Department of Otolaryngology. The study was approved by our hospital Ethics Committee of Yiwu central hospital. The inclusion criteria were as follows: adult unilateral FMS, no obvious bone destruction on computed tomography (CT), and CT confirmation that all lesions were confined to the maxillary sinus. The exclusion criteria were as follows: evidence of irregular bone destruction or involvement of another nasal sinus on CT, histological evidence of a malignant tumor, chronic sinusitis or odontogenic sinusitis, and revision surgery. We recorded patient age, sex, lesion side, active smoking status (more than 5 cigarettes per day, the parameter pack/years has been reported in the medical history), as well as the surgical procedure(s) performed, recurrence, complications, and follow-up duration.

### Surgical procedure

All patients were placed supine with the head slightly elevated; hypotensive general anesthesia was then induced. Cotton mixed with decongestants was inserted into the nose 10 min before surgery. All procedures were performed by experienced surgeons using rigid 0°, 30°, or 70° 4-mm endoscopes (Hangzhou Tonglu Apex Endoscope Co. LTD, China). No patient was treated via the Caldwell-Luc approach. The criteria for performing an extended MMA included the thin medial wall or bone defect by CT but the thicker bone wall and only the inferior wall in combined approach.

### Extended MMA

The uncinate process was removed following debridement of the polypoid mucosa, and a wide MMA was created by removing most of the posterior fontanelle and connecting a possible secondary maxillary ostium to the area of the maxillary natural ostium anteriorly. In addition, when the fungus ball was located on the anterior or inferior side of the MS, the MMA was further enlarged via extended medial wall, which had been destroyed or became thin in most of cases. The retrogradation removal of medial mucosa was performed to clearly expose all the wall as possible as. After widening of the antrostomy, the fungus ball was completely extracted using a suction tube, and curved microdebrider blades, then, the MS irrigation was applied using 1% povidone iodine solution. Care was taken to avoid any removal of MS mucosa.

### MMA combined with IMA (Combined approach)

**Fig. 1:**
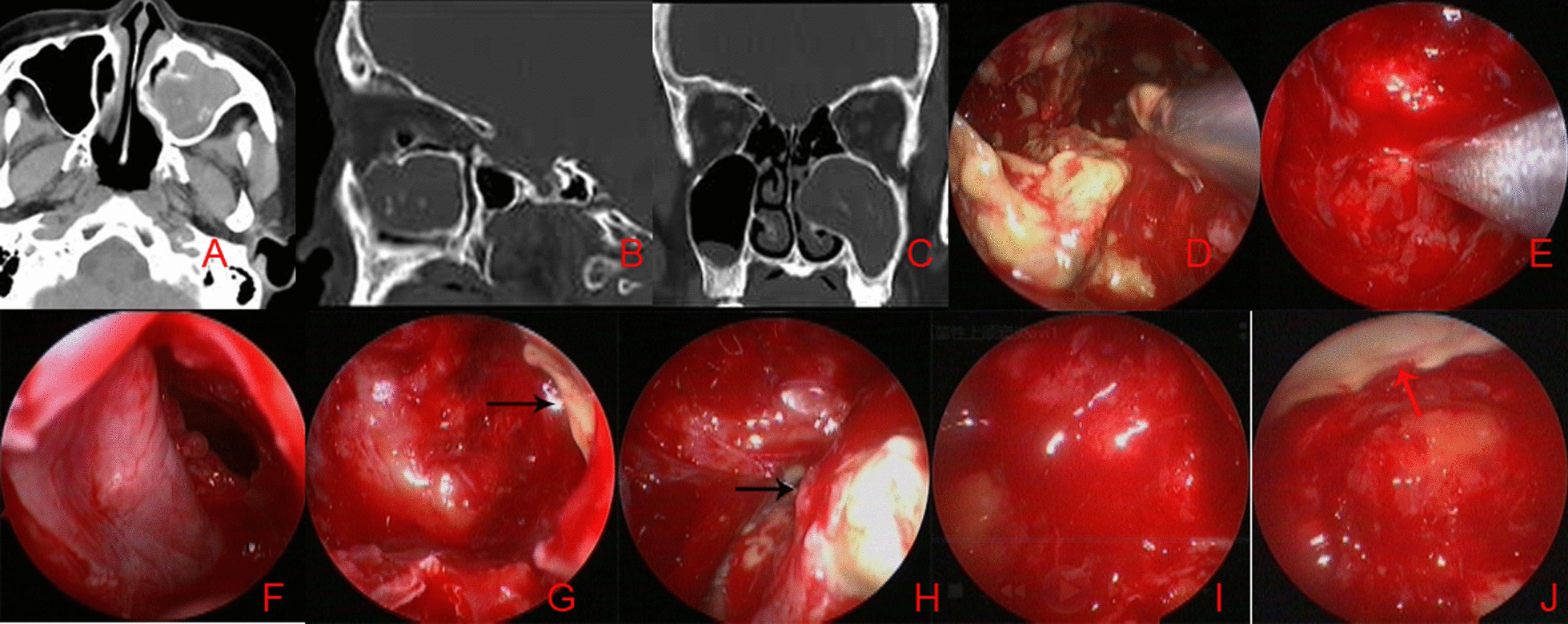
Combined approach was performed. Preoperative CT revealed FMS (**A**–**C**); fungus blocks were removed through MMA (**D**). No additional fungus block was revealed via MMA (**E**). IMA (**F**) revealed a residual fungus block in the alveolar recess (black arrows). The residual fungus block was found (**G** and **H**) and removed through IMA (**I**), but revealed another block in the anterior wall (red arrows) (**J**)

The inferior turbinate was gently medialized using a Freer elevator following standard MMA. The inferior opening of the nasolacrimal duct (NLD; the Hasner valve) was identified to avoid any injury to the lacrimal pathway. The meatal flap was elevated on the inferior and lateral mucosa in the inferior meatus. After elevation from the meatal bone, the flap was positioned on the nasal floor and an inferior meatus window was then created using a perforator. The bony wall was removed using a bone drill to make a sufficiently wide opening in the MS. The bridge of bone between the two antrostomies preserved the inferior turbinate. If the lesion was located in the anterior, inferior or medial regions, the microdebrider blades were passed through the inferior antrostomy using a 70° endoscope. Nasal and sinus 1% povidone iodine solution and saline irrigation were performed, the mucosal flap was positioned and the inferior turbinate was then lateralized, ensuring complete hemostasis (Fig. 1).

### Postoperative management

All patients underwent nasal packing with Merocel [Medtronic Xomed, Jacksonville, Fla.], and all specimens were sent to the Department of Pathology. Nasal packing was removed on the second postoperative day; daily saline nasal spray was then performed. Topical or systemic steroids (methylprednisolone, 20 mg per day) were prescribed if edema of the MS mucosa was detected during the first follow-up visit (1 month after surgery). Postoperative complications included facial swelling, upper lip numbness, and antrostomy closure. Postoperative follow-up endoscopy was easily scheduled 1, 6, 12, and 36 months after surgery for all patients. In addition, postoperative CT was performed 12, and 36 months after surgery.

### Statistical analysis

Data are expressed as means (with standard deviations) for quantitative variables and as frequencies (with percentages) for qualitative variables. Between-group comparisons were made using the independent-samples t-test for quantitative variables and the Chi-square test for qualitative variables. All statistical analyses were performed using SPSS version 20 (IBM, Armonk, NY, USA). P < 0.05 was considered to indicate statistical significance.

## Results

### Demographic data

In total, 90 patients with FMS were included in the analysis. Of the 90 patients, 52 patients were in the extended MMA group and 38 patients in the combined approach group.The average age, affected side, and sex were matched among two groups (Table 1). The average operation time of the combined approach group was significantly longer than in the extended MMA groups (Table 1). The anatomical variations of OMC was found in 68 (75.6%) patients in this study, of the 68, concha bullosa was in 21.1% patients, narrow infundibulum in 42.4%, and variations of uncinate process in 34.8%. CT revealed the thin medial wall or bone defect in 57 (63.33%) patients.

**Table 1:** Demographic data and average operation time among two groups

	Extended MMA (n = 52)	Combined approach (n = 38)	*P* value
Average age, years	47.6 ± 5.4	47.9 ± 6.9	0.884^b^
Sex (F:M)	38:14	26:12	0.805^a^
Active smoking status(Y:N)	4:48	3:35	0.716^a^
The side (L:R)	31:21	21:17	0.844^a^
Average operation time, minutes	42.5 ± 6.5	57.4 ± 4.9	< 0.01^b^

^a^Chi-square test

^b^Independent samples test

### Recurrence rate and complications

The mean operation time in the extended MMA group was significantly shorter than that of combined approach group (42.5 ± 6.5 vs 57.4 ± 4.9, Independent Samples Test, P < 0.01). At postoperative 12 months postoperatively, the recurrence rate was 3.85% (2/52) in the extended MMA group and 0.0% (0/38) in the combined approach group, the difference wasn’t significant (*X*^*2*^ = 0.618, P > 0.05). The recurrence rate wasn’t increased during the follow-up period over time in both groups.

13.5% (7/52) patients complained of cheek numbness in the extended MMA group, 60.5% (23/38) patients complained of cheek numbness and epiphora in 5.3% (2/38) patients in the combined approach group, the difference was significant (*X*^*2*^* test*, P < 0.01). However, no major complications were observed in both groups. In addition, IMA closure was observed in 4 (10.5%) in the combined approach group at 12 months postoperatively and in 9(23.6%) at 36 months postoperatively.

## Discussion

ESS with MMA was the gold standard surgical technique for FMS. Unfortunately, rhinologists subsequently found that endoscopic MMA alone is limited in terms of visualizing the whole MS cavity, and this incomplete access may lead to a recurrence of FMS [6, 12]. Some studies have reported FMS recurrence rates of 3–14% after MMA alone [6, 13]. The high recurrence rates of MMA alone is related to the anatomical structure of MS. It may be difficult to examine the anterior inferior or medial inferior wall through the standard MMA site, even when using a 70° endoscope. Therefore, a fungus ball located in the anterior inferior and medial inferior wall of MS might be incompletely removed through a middle meatal window [13]. However, visualization of the whole sinus are key to the complete removal of fungal debris. This suggests the need for another approach that would yield intraoperative benefits. IMA allows visualization of the entire MS, especially the anterior inferior and medial inferior walls [13]. Choi et al. [12] observed using fiber-optic sinus exam that the postoperative residual fungal debris was found in 9.5% and 29.2% of in the Combined approach and MMA alone groups.

In this study, the recurrence rate was 3.85% (2/52) in the extended MMA group and 0.0% (0/38) in the combined approach group (P > 0.05). In addition, the recurrence rate wasn’t increased over time in both groups. We performed the extended MMA via extended medial wall in this study, thereby formed a very wide MMA antrostomy, which clearly exposed all the wall of MS as possible. In a way, the extended MMA was similar to standard MMA combined with IMA. We found the thin medial bone wall or bone defect in 57 (63.33%) patients, retrogradation removal wasn’t difficult using curved microdebrider blades.

Another strength of extended MMA is not only to avoid the postoperative complications but also correct the anatomical variations. Previous study had suggested that the anatomical variations of OMC was important cause of fungal growth, if it weren’t corrected, FMS had high possibility of recurrence [14]. However, the anatomical variations of OMC may be completely corrected through extended MMA. In addition, our and other studies found high IMA window closure rate for the patients with IMA [12]. Previous study suggested that the mucociliary clearance was toward all along the MS ostium even if IMA was performed [15], thus we believed that extended MMA via extended medial wall was enough but IMA wasn’t necessary for the treatment of FMS in most cases.

## Conclusions

This study suggested that extended MMA via antidromic extended medial wall of MS may effectively prevent the recurrence of FMS and reduce the complications, IMA wasn’t necessary for the treatment of FMS in most cases.

## Data Availability

All data generated or analyzed during this study are included in the published article.
